# Machine learning algorithms to predict khat chewing practice and its predictors among men aged 15 to 59 in Ethiopia: further analysis of the 2011 and 2016 Ethiopian Demographic and Health Survey

**DOI:** 10.3389/fpubh.2025.1555697

**Published:** 2025-03-27

**Authors:** Mequannent Sharew Melaku, Lamrot Yohannes, Eliyas Addisu Taye, Nebebe Demis Baykemagn

**Affiliations:** ^1^Department of Health Informatics, Institute of Public Health, College of Medicine and Health Sciences, University of Gondar, Gondar, Ethiopia; ^2^Department of Environmental and Occupational Health and Safety, Institute of Public Health, College of Medicine and Health Science, University of Gondar, Gondar, Ethiopia

**Keywords:** predictors, khat chewing practice, prediction, machine learning algorithms, demographic health survey, Ethiopia

## Abstract

**Introduction:**

Khat chewing is a significant public health issue in Ethiopia, influenced by various demographic factors. Understanding the prevalence and determinants of khat chewing practices is essential to developing targeted interventions. Therefore, this study aimed to predict khat chewing practices and their determinant factors among men aged 15 to 59 years in Ethiopia using a machine learning algorithm.

**Methods:**

This study used data from the 2011 and 2016 Ethiopian Demographic and Health Surveys (EDHS). A weighted sample of 26,798 men aged 15 to 59 years was included in the study. STATA version 17 was used for data cleaning, weighting, and descriptive statistical analysis. Python 3.12 software was used for machine learning-based predictions of khat chewing among men. Furthermore, Decision Tree, Logistic Regression, Random Forest, KNN, Support Vector Machine, eXtreme Gradient Boosting (XGBoost), and AdaBoost classifiers were employed to identify the most critical predictors of khat chewing practices among men. In addition, accuracy and the area under the curve were used to evaluate the performance of predictive models.

**Result:**

From a total weighted sample of 26,798 men, 8,786 men (32.79) aged 15 to 59 years reported chewing khat. The eXtreme Gradient Boosting (XGBoost) model demonstrated the highest predictive accuracy at 87%, with an area under the ROC curve (AUC) of 0.94. The Beeswarm plot from the SHAP analysis (based on the XGBoost classifier model) identified the top-ranked variables for predicting khat chewing among men, including age, religion, region, wealth index, age at first sexual encounter, frequency of watching television, frequency of listening to the radio, and number of sexual partners.

**Conclusion:**

Overall, three in 10 men in Ethiopia chew khat. The XGBoost model demonstrated superior predictive performance in identifying the determinants of khat chewing practices. This model identified age, religion, region, wealth index, age at first sexual encounter, media exposure, and the number of sexual partners as key predictors of khat chewing among Ethiopian men. Effective khat prevention strategies should focus on the following: preserving rural norms that discourage khat use and expanding these to urban areas; targeted interventions for young and middle-aged men, including youth programs and economic empowerment initiatives as alternative opportunities; strengthening family values through marriage counseling and spouse involvement to help reduce khat chewing; integrating khat education into reproductive health programs and engaging religious leaders in awareness efforts; and, finally, implementing media campaigns, school-based education, and policy measures—such as restricting sales near schools and enforcing community bylaws—to further curb khat consumption while promoting healthier economic alternatives.

## Introduction

Globally, an estimated 5 to 20 million people engage in khat chewing practices, with the majority residing in the Horn of Africa and the Arabian Peninsula. Khat chewing is a widespread practice primarily in these regions, carrying significant cultural and economic implications. In Yemen, khat is deeply ingrained, with 60–90% of men and 35% of women chewing it daily, occupying nearly 40% of agricultural land and contributing to severe water shortages. Djibouti and Somalia also report high prevalence rates, particularly among men, where khat chewing plays a key role in social interactions and generates substantial revenue. In Kenya, khat, known as “miraa,” is a legal cash crop that significantly contributes to the economy through exports. Conversely, in Saudi Arabia, khat is illegal; however, large quantities are smuggled in, mainly from Yemen. Overall, khat chewing remains widespread in regions where it maintains cultural and economic significance, despite concerns over its health effects and legal restrictions in several countries (1) https://en.wikipedia.org/wiki/Khat.

Khat (*Catha edulis*) is a plant extensively cultivated in the Horn of Africa. People chew its leaves for their stimulating properties due to the presence of psychoactive substances such as cathinone and cathine, which induce stimulant effects upon chewing (2). The chewing of khat has been a persistent global public health concern due to its association with various health risks, socioeconomic implications, environmental impacts, and reduced work effectiveness among the younger population (3–5). Studies indicate that regular consumption may lead to adverse health effects, including elevated blood pressure, increased heart rate, gastrointestinal issues, and psychological dependence (4).

Chewing khat is common throughout Africa, especially among the countries that comprise the Horn of Africa, including Ethiopia (4). Over 20 million individuals chew khat every day worldwide, with East Africa and the Arabian Peninsula being the regions where this practice is most prevalent (6). Consequently, a systematic review study showed the prevalence rates of 90% in Yemen, 90% in Djibouti, and 88% in northwestern Kenya were observed (7). According to the World Health Organization (WHO) (2), it has multiple effects on individuals, organizations, and society at large. These effects include adverse health outcomes and social and economic consequences (8).

Health issues associated with khat use include dental problems, mental health issues, digestive disorders, and an increased risk of heart disease (9). Additionally, chronic khat use is associated with specific health conditions such as insomnia, anxiety, depression, and loss of appetite (10). Among these issues, sleep disturbances are particularly prevalent, accounting for 65% (11). Moreover, it contributes to psychological dependence and addiction (12). Furthermore, khat has a major and detrimental social and economic impact, resulting in a rise in criminal activity (11%), a reduction in overall productivity (30%), and an increase in absenteeism at work (18%) (13). Khat chewing can also lead to addiction and psychological dependence, as well as physical health risks such as increased heart rate and blood pressure, gastrointestinal issues, oral health problems, and psychiatric effects (9). In Saudi Arabia, a study found that 29.3% of diabetes mellitus cases could be linked to khat chewing, among other factors (14). According to the evidence, the primary predictors of khat chewing include gender (specifically being male), religious beliefs, early age of initiation, having colleagues who chew khat, and exposure to alcohol and cigarette use, as well as having family members who chew khat (4, 15). Recent studies indicate that men are seven times more likely than women to chew khat, with an increasing trend observed over time, rising from 13 to 24.2% (4).

In Africa, particularly in Kenya and Ethiopia, the prevalence of khat chewing is higher among men, with rates of 54.8 and 22.6%, compared to women at 36.8 and 9.1%, respectively (16, 17). Moreover, evidence from various regions of Ethiopia indicates that khat chewing is common among adult men and is associated with an increased risk of male sexual impotence and sexually transmitted infections (5, 18–21).

Previous studies were conducted in small study areas using traditional analysis models. Some studies have been conducted using the demographic and health survey dataset of Ethiopia; however, these studies used traditional analysis methods to identify determinant factors. Earlier research primarily relied on logistic regression and other conventional statistical techniques. Machine learning offers improved predictive performance and can capture complex nonlinear relationships among multiple predictors. As practical studies have shown, machine learning provides powerful tools for analyzing complex datasets and identifying patterns that traditional statistical methods may not reveal (22). This study aimed to predict various factors influencing men’s khat chewing practices in Ethiopia from 2011 to 2016 through the application of machine learning techniques. This approach allows for a more comprehensive investigation of the predictors that influence khat consumption, leading to more precise forecasts and prioritized actions.

## Materials and methods

### Study setting

The study was conducted in Ethiopia, which is located in the Horn of Africa. Ethiopia has nine administrative regions (Tigray, Afar, Amhara, Oromia, Somalia, Benishangul Gumuz, Gambella, Harari, and SNNPR) and two city administrations, Dire Dawa and Addis Ababa. These regions are divided into 68 zones, 817 districts, and 16,253 kebeles. This study used the 2011 and 2016 EDHS data, which were conducted from 18 January 2011 to 27 June 2011 and 18 January 2016 to 27 June 2016, respectively.

### Population

All men in Ethiopia aged 15 to 59 years were considered as both a source and a study population. A total of 26,798 men who met the inclusion criteria from this age group were included in the overall analysis. A stratified, two-stage cluster sampling technique was employed for the 2011 and 2016 EDHS to select study participants. The data were obtained from the DHS Program’s website^1^ after being authorized to utilize the data.

### Dependent variable

The outcome variable, khat chewing practice, was measured dichotomously as “Yes/No.” Participants’ self-reported answers during the survey indicated their khat chewing practice.

### Independent variable

Sociodemographic factors (including age, educational level, literacy, marital status, employment status, wealth index, and sex of the household head), residence, region, social media usage (such as newspaper reading habits, radio listening, and television watching), age at first sexual encounter, and the number of living children.

### Data management and analysis

**Figure 1:**
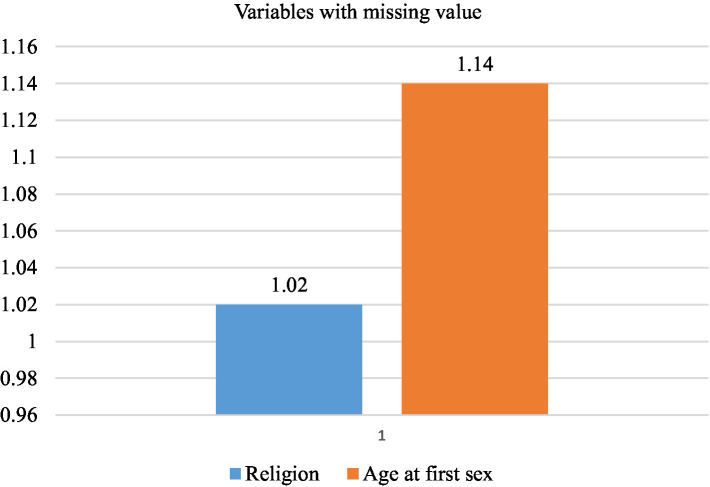
A graph illustrating the variables with the percentage of missing values.

STATA version 17 and Microsoft Excel 2019 were used to clean and weigh the data. Descriptive and summary statistics were calculated using STATA version 17 software. Python version 3.9 was used for data processing and analysis, featuring significant packages, including Pandas, Scikit-learn, Imbalanced-learn, SHAP, and Apriori. The seven steps of the machine learning framework, which include data collection, preparation, model selection, training, evaluation, parameter tuning, and prediction, were conducted during the machine learning analysis. The study used data from the 2011 and 2016 Ethiopian demographic health surveys to train a machine-learning model for accurate predictions. The data underwent multiple preprocessing steps, including handling missing values, normalization, standardization, categorical encoding, feature selection, dimensionality reduction, and addressing class imbalance. Missing values were addressed using the k-nearest neighbors’ imputation method with k = 5. The distribution of missing values in the dataset is illustrated in Figure 1. Nominal categorical variables were encoded using one-hot encoding. Feature scaling was performed using both Min-Max normalization and standardization (z-score normalization), where Min-Max scaling transformed features to a specific range, while standardization rescaled features to have a mean of zero and a standard deviation of one.

The model’s performance improved by selecting relevant features using the Recursive Feature Elimination method. The dataset was balanced using the Synthetic Minority Over-Sampling Technique (SMOTE) before training to ensure unbiased results. Seven algorithms were used, integrating supervised learning techniques. Feature importance analysis was conducted to identify predictors, and relevant rules were extracted from the best-performing model. The classification algorithms used for this analysis included AdaBoost Classifier, XGB Classifier, Random Forest (RF), K-Nearest Neighbor classifier (KNN), Light Gradient Boosting classifier (LGBM), Extreme Gradient Boosting (XGBoost), and Decision Tree. These algorithms were selected based on prior research that employed machine learning methods for task classification.

The entire dataset was divided into training and testing sets by randomly assigning 80% of the data for model training and 20% for testing the trained model. The common k-fold cross-validation methodology was used to ensure the model’s performance because the train-test split method can lead to overfitting or underfitting. The k-fold approach divides the dataset into ‘K’ sub-samples, using one for testing and the remaining for training, and this process is repeated K times.

Thus, the 10-fold cross-validation performance measure is the average of the values calculated in this loop (23). In this study, we utilized stratified 10-fold cross-validation to ensure that each fold maintains the class distribution. This method helps preserve the proportion of each class in the training and validation sets, which is particularly important for imbalanced datasets. The outcome variable consisted of two mutually exclusive categories related to khat chewing practices; therefore, the dataset used in the analysis fell under the category of binary classification. The models’ performance was evaluated using accuracy, the area under the curve (AUC), precision, recall, and F1 score. These metrics provided a comprehensive evaluation of the models’ accuracy, their ability to correctly classify instances, and their overall predictive power.

### Shapley additive explanations

Model interpretability was rigorously examined using SHAP (SHapley Additive exPlanations), a game-theoretic framework that quantifies feature contributions to machine learning predictions. This study employed two principal metrics: the mean absolute SHAP value, reflecting the average magnitude of a feature’s influence across all instances, and the mean SHAP value, capturing the net directional impact of features on model outputs (24–26). The link between the predictors and the outcome variable was assessed using the Additive Explanations (SHAP) feature significance approach, which also assisted in identifying the independent factors that are most important for predicting the Zero status of children (27). SHAP analysis uses a game theory framework to provide a global or local interpretation and explanation for any machine learning model’s prediction (27).

In our analysis, we selected SHapley Additive exPlanations (SHAP) over Recursive Feature Elimination (RFE) due to SHAP’s ability to provide both global and local interpretability by quantifying each feature’s contribution to the model’s predictions. While RFE removes less important features iteratively based on model performance, SHAP offers deeper insights into feature importance and interactions, making it particularly useful for understanding complex relationships within the data. Additionally, SHAP values are based on cooperative game theory and provide a unified measure of feature importance, which helps break down a prediction to show the impact of each feature (28).

### Association rule mining

Association rule mining is among the most important and popular data mining techniques. It is used to discover hidden patterns and relationships based on specific confidence intervals and lift, thereby addressing limitations in feature selection (29).

### Ethical consideration

The study involved a secondary data analysis utilizing publicly available DHS data, making ethical approval and participant consent unnecessary. The IRB-approved procedures for DHS public-use datasets ensure that respondents, households, or sample communities cannot be identified in any way. The data files do not contain names of individuals or household addresses. Under the MEASURE DHS Program, respondents’ privacy is well-protected. The confidentiality of respondents is protected by the MEASURE DHS Program. The MEASURE DHS Program website (see footnote 1) is where we found the dataset. Furthermore, all the materials used for this research were appropriately acknowledged.

## Results

### Regional distribution of khat chewing practice

This study included a total of 26,798 weighted participants. The largest number of participants came from the Oromia region, with 5,096; the Amhara region, with 4,589; and SNNPR, with 4,278. Conversely, the smallest numbers of participants were from the Harari region, with 681; Diredawa, with 902; and Gambela, with 868. Overall, the khat chewing practice among men is 32.79 percent, ranging from 733 (15.97) in the Amhara region to 511 (75.04) in the Harari region. Among the participants, the regions with the highest numbers of khat chewers in Ethiopia were Harari, with 511 (75.04); Diredawa, with 573 (63.53); Oromia, with 2,143 (42.05); and SNNPR, with 1,469 (34.34) (Table 1).

**Table 1:** Regional distribution of study participants with khat chewing practices.

Region	No	Yes	Weighted sample	Khat chewing practice (yes) weight
Tigray	2,715 (75.97)	859 (24.03)	3,574 (13.30)	9.80
Afar	1,145 (66.15)	586 (33.85)	1,731 (6.50)	6.70
Amhara	3,856 (84.03)	733 (15.97)	4,589 (17.10)	8.30
Oromia	2,953 (57.95)	2,143 (42.05)	5,096 (19.0)	24.40
Somalia	858 (49.68)	869 (50.32)	1,727 (6.40)	9.90
Benshangul	1,693 (79.86)	427 (20.14)	2,120 (7.90)	4.90
SNNPR	2,809 (65.66)	1,469 (34.34)	4,278 (16.0)	16.70
Gambela	654 (75.35)	214 (24.65)	868 (3.20)	2.40
Harari	170 (24.96)	511 (75.04)	681 (2.50)	5.80
Addis Ababa	830 (67.37)	402 (32.63)	1,232 (4.60)	4.60
Diredawa	329 (36.47)	573 (63.53)	902 (3.40)	6.50
Total	18,012 (67.21)	8,786 (32.79)	26,798	100.0

### Sociodemographic characteristics

The majority of participants, 9,664 (36.06%), were aged 15 to 24 years, followed by those aged 25 to 34 years, totaling 7,571 (28.25%). Approximately 18,716 (69.84%) of the participants resided in rural areas. Similarly, the majority, 15,861 (59.19%), of the participants had primary and secondary education levels (Table 2).

**Table 2:** Sociodemographic characteristics of men aged 15 to 59 years in Ethiopia.

Variables	Frequency
Age	
15–24	9,664 (36.06)
25–34	7,571 (28.25)
35–44	5,381 (20.08)
45 and above	4,182 (15.61)
Religion	
Orthodox	11,602 (43.30)
Catholic	242 (0.90)
Protestant	4,326 (16.14)
Muslim	10,182 (38.00)
Traditional	443 (1.65)
Residence	
Urban	8,082 (30.16)
Rural	18,716 (69.84)
Education	
No education	7,966 (29.73)
Primary	12,002 (44.79)
Secondary	3,859 (14.40)
Higher	2,971 (11.09)
Sex of household head	
Male	22,880 (85.38)
Female	3,918 (14.62)
Literacy	
Unable to read/write	8,740 (32.61)
Able to read/write	18,058 (67.39)
Wealth Index	
Poor	9,674 (36.10)
Middle	3,893 (14.53)
Rich	13,231 (49.37)
Marital status	
Single	11,449 (42.72)
Married	15,349 (57.28)
Occupation	
Not Working	2,568 (9.58)
Working	24,230 (90.42)
Read newspaper	
Yes	9,563 (35.69)
No	17,235 (64.31)
Listening Radio	
Yes	17,313 (64.39)
No	9,485 (35.39)
Watching Television	
Yes	15,456 (57.68)
No	11,342 (42.32)
Age at first sexual encounter	
Not had sex	7,796 (29.09)
15–19	6,434 (24.01)
20–24	4,942 (18.44)
More than 25	2,280 (8.51)
At first union	5,346 (19.95)
Number of living children	
1	14,972 (55.87)
2–4	6,398 (23.87)
>= 5	5,428 (20.26)

### Machine learning analysis

#### Compression of the proposed model

**Figure 2:**
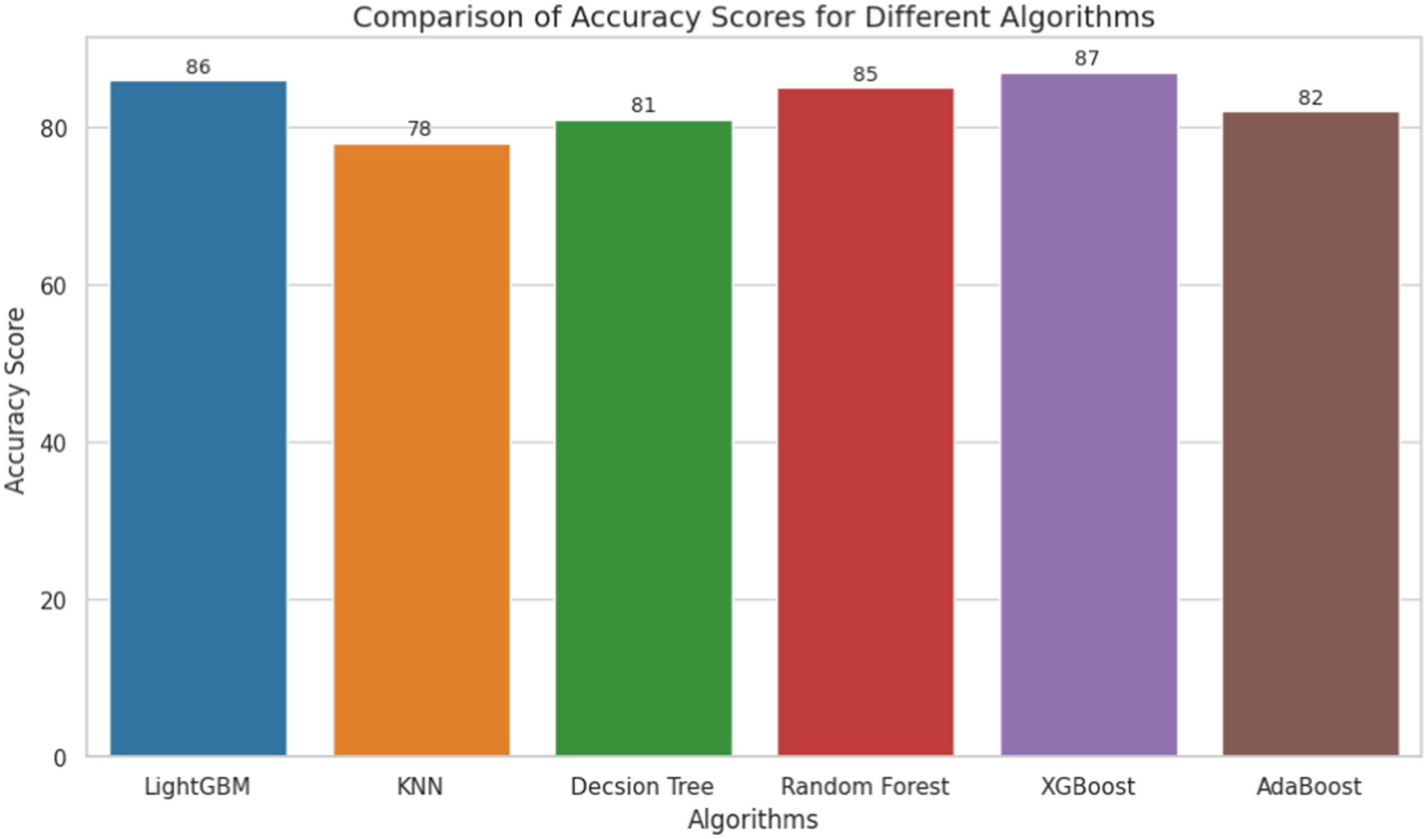
Accuracy of machine learning models for khat chewing practices in Ethiopia from 2011 to 2016.

**Figure 3:**
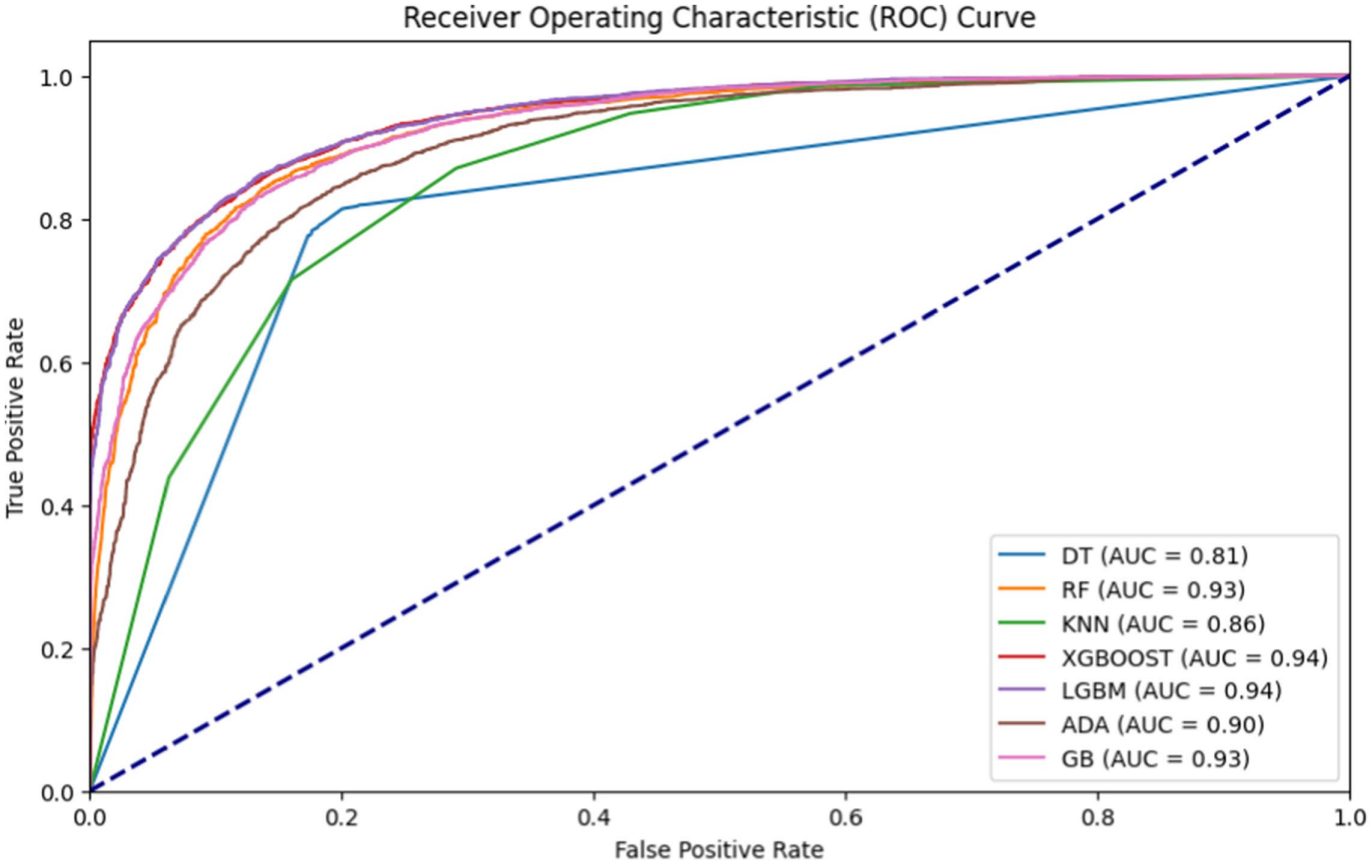
AUROC of machine learning models used for khat chewing practices in Ethiopia from 2011 to 2016.

To assess the predictive ability of the models regarding khat chewing practices, the accuracy, AUC, precision, recall, and F1 scores of the ML models were compared. This comparison was performed using an 80:20 train-test split, with 80% of the data used for training and 20% for testing purposes. To avoid biased model building, the comparison of the ML models was conducted after balancing the training data using the SMOTE oversampling method. After comparing the ML models, the XGBoost classifier emerged as the best predictive model, achieving an accuracy of 87%. It achieved an accuracy of 87%, an AUC of 94, a precision of 86, a recall of 85, and an F1 score of 86. Furthermore, the accuracy of the model was reevaluated through 10-fold cross-validation, as this method provides a more rigorous assessment of model performance. Unlike train-test splitting, which can be sensitive to specific data points, cross-validation divides the dataset into multiple training and testing sets. This approach helps to reduce the impact of any particular split and ensures that randomness does not influence the performance of the model. After evaluating the ML models using cross-validation, the XGBoost algorithm achieved a comparable accuracy of 87%, as observed in the train-test split (Table 3 and Figures 2, 3).

**Table 3:** Accuracy, AUC, precision, recall, and F-measure for the proposed machine learning models.

Machine learning models	Dataset	Accuracy	AUC	Precision (Sensitivity)	Recall	F1-Score
KNN Classifier	Before SMOTE (%)	74	78	91	66	69
	After SMOTE (%)	78	86	75	85	79
LGBM classifier	Before SMOTE (%)	82	89	75	68	71
	After SMOTE (%)	86	94	86	85	86
Decision Tree Classifier	Before SMOTE (%)	75	73	64	60	62
	After SMOTE (%)	81	82	81	80	81
Random Forest Classifier	Before SMOTE (%)	80	86	72	66	69
	After SMOTE (%)	85	93	85	85	85
XGBoost classifier	Before SMOTE (%)	81	88	74	69	71
	After SMOTE (%)	87	94	86	85	86
AdaBoost Classifier	Before SMOTE (%)	81	87	76	63	69
	After SMOTE (%)	82	90	80	84	82

#### Importance of feature selection

**Figure 4:**
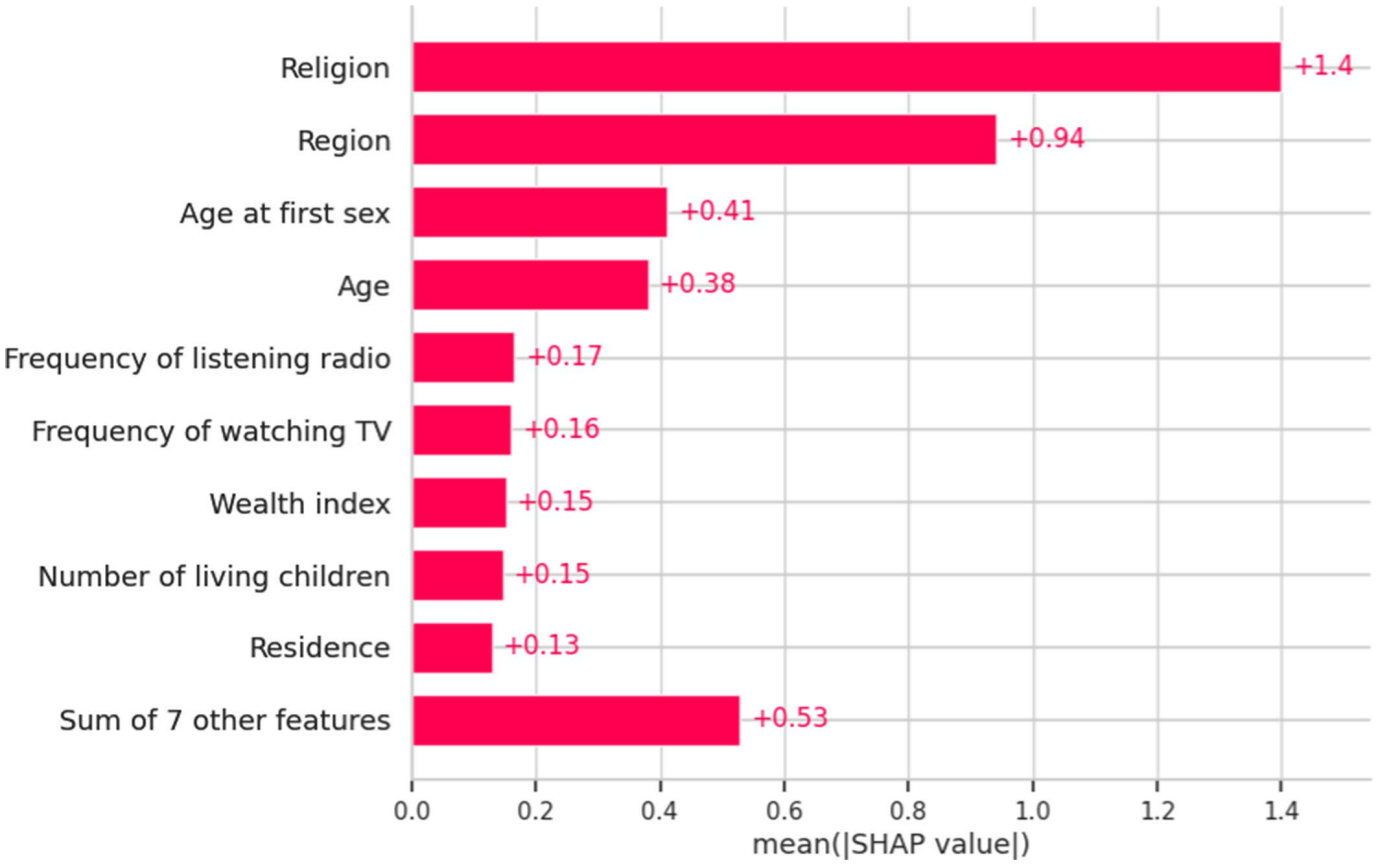
Important features identified by light gradient boosting and their SHAP values regarding the determinants of khat chewing practices in Ethiopia from 2011 to 2016.

The importance of feature selection lies in reducing the cost of learning by decreasing the number of features. In this study, we deploy wrapper methods using SHAP values. The wrapper algorithm method was employed to identify the most significant factors related to khat chewing practices. We selected important features based on light gradient boosting to narrow down the set of potential features shown in Figure 4.

#### Beeswarm plot

The beeswarm plot provides valuable insights into the relationship between features and the target variable. Each point in the plot represents a feature and its corresponding SHAP value, illustrating the impact of each feature on the likelihood of khat chewing practice. The position of the points relative to the vertical line at the “0” SHAP value indicates the impact of the feature on the likelihood of khat chewing practice. On the right side of the vertical line, where the SHAP values are positive, features increase the likelihood of khat chewing practices.

The red line represents Category “1” (i.e., “NO” khat chewing practices or a high value of the target variable), suggesting that increasing the values of these features tends to increase the predicted value of the target variable.

For example, increasing the values of region, religion, age at first sexual encounter, age, frequency of listening to the radio, frequency of watching television, education, wealth index, the number of living children, and residence had a significant positive impact on predicting khat chewing practice toward Category “1.” Conversely, when these factors decrease, they have a corresponding influence toward Category “0.”

**Figure 5:**
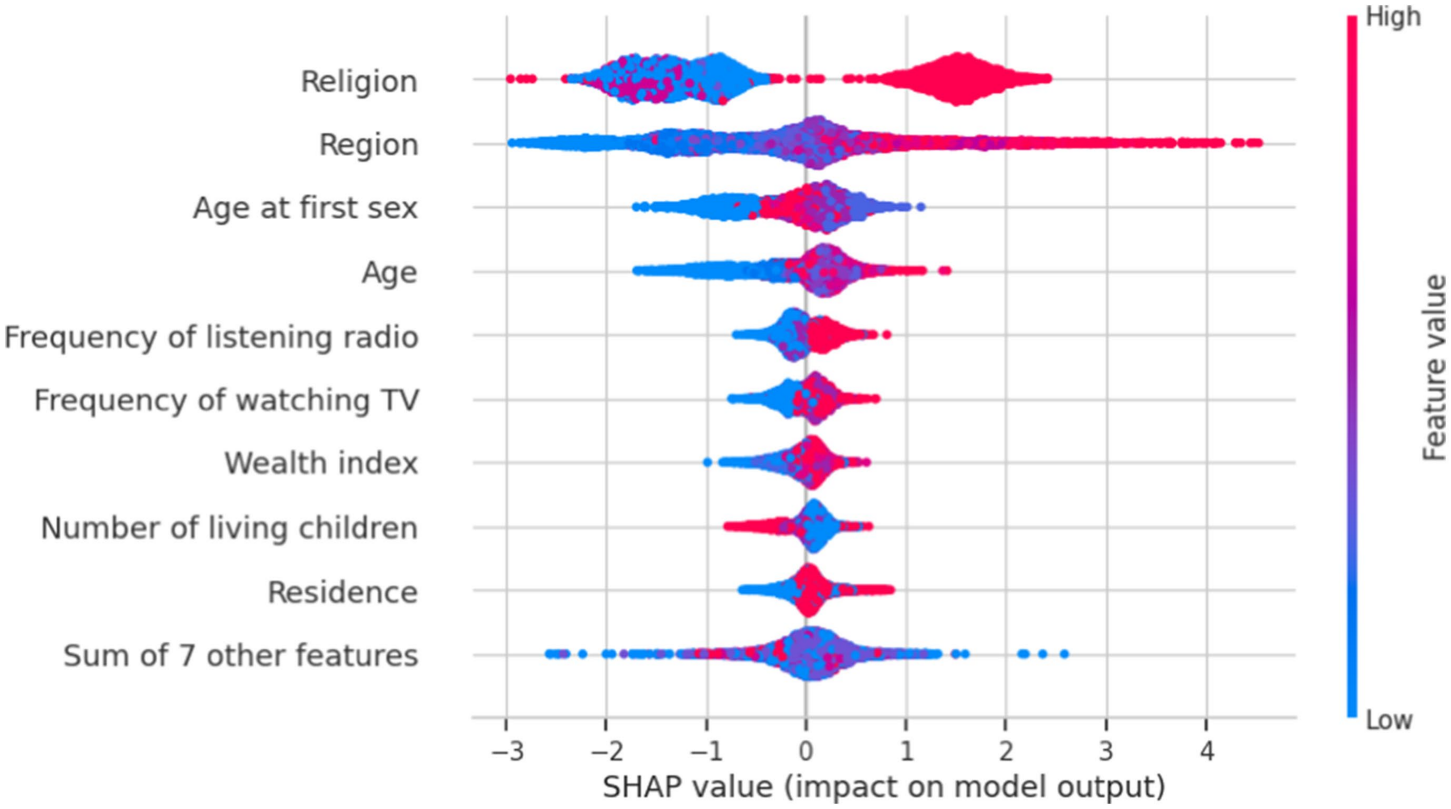
Important features of SHAP value impact on the model for khat chewing practices in Ethiopia from 2011 to 2016.

On the left side of the vertical line, where SHAP values are negative, the features are associated with a decreased likelihood of non-khat chewing practices (class 1). This is depicted by the blue line, which represents the Category “0” (i.e., khat chewing practices). Increasing the value of these features generally leads to a decrease in the predicted value of the target variable toward Class 1 (non-khat chewing practices) (Figure 5).

#### Rule extraction

Rule 1: This rule states that if a participant is uneducated and practices an orthodox religion, the likelihood of not chewing khat is quite high (91.7% confidence). The lift value of 1.39 indicates that this association is significantly stronger than what would be expected by chance.

Rule 2: This rule states that if a participant is married, resides in a rural area, and practices an orthodox religion, the probability of not chewing khat is high (89.96% confidence). The lift value of 1.39 indicates that this association is significantly stronger than expected by chance.

Rule 3: This rule states that if a participant is from a rural area and practices an orthodox religion, the probability of not chewing khat is high (with 88.66% confidence). The lift value of 1.37 indicates that this association is significantly stronger than would be expected by chance.

Rule 4: This rule states that if a participant resides in a rural area, is literate, and practices an orthodox religion, the probability of not chewing khat is high (87.89% confidence). The lift value of 1.36 indicates that this association is significantly stronger than expected by chance.

Rule 5: This rule states that if a participant does not watch television and practices the Orthodox religion, the probability of not chewing khat is high (with 87.66% confidence). The lift value of 1.36 indicates that this association is significantly stronger than expected by chance.

Rule 6: This rule states that if a participant is a rural resident who practices the Protestant religion, the probability of not chewing khat is high (85.5% confidence). The lift value of 1.33 indicates that this association is significantly stronger than would be expected by chance.

Rule 7: This rule states that if a participant does not listen to the radio and practices an orthodox religion, the probability of not chewing khat is high (with 85.03% confidence). The lift value of 1.32 indicates that this association is significantly stronger than would be expected by chance.

Rule 8: This rule states that if a participant is married and identifies as Protestant, the probability of not chewing khat is high (84.38% confidence). The lift value of 1.21 indicates that this association is significantly stronger than what would be expected by chance.

**Figure 6:**
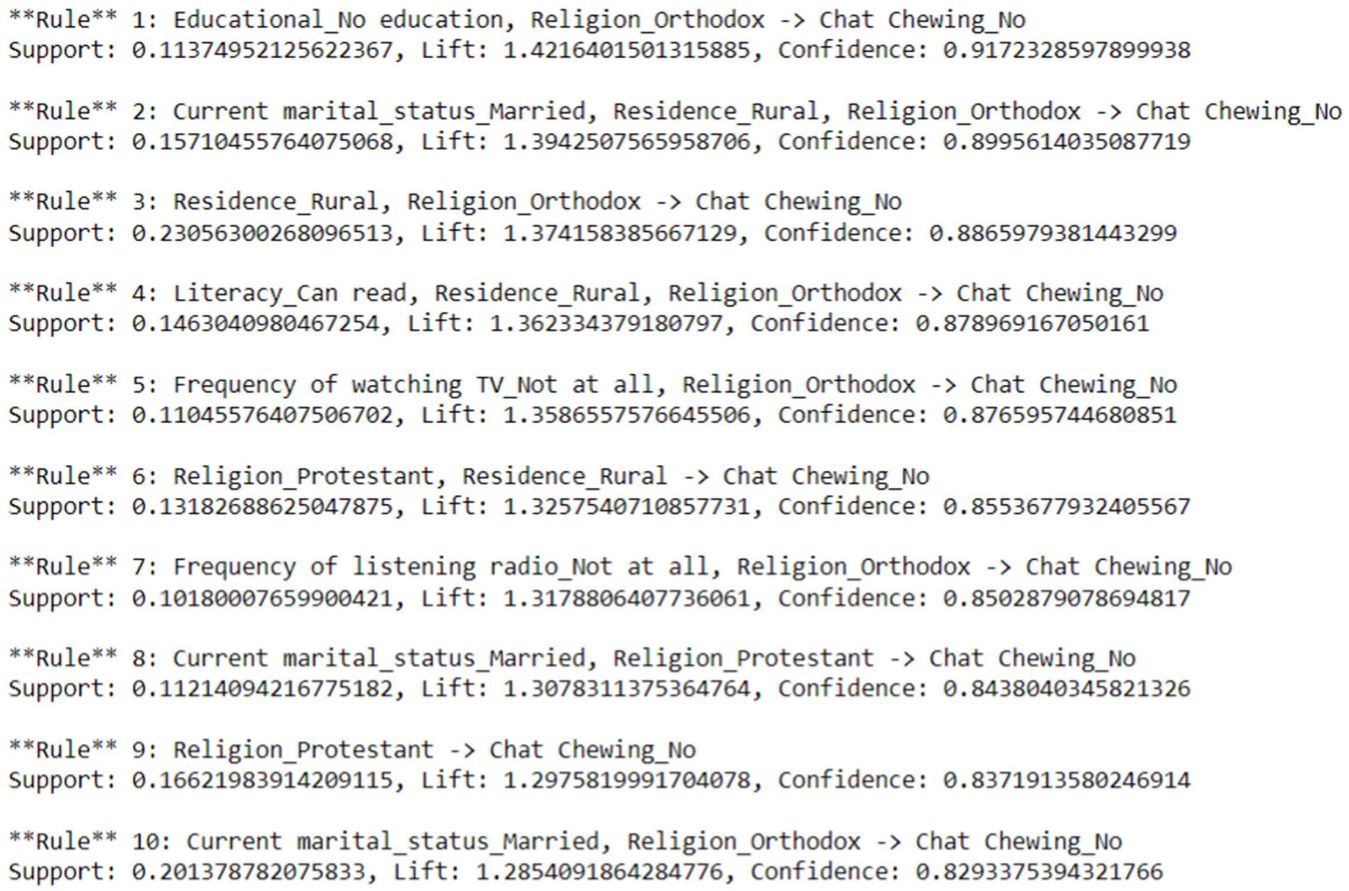
Rule extraction for the determinants of khat chewing practices in Ethiopia from 2011 to 2016.

Generally, the association rules indicate a higher probability of not chewing khat among participants from rural areas, those practicing Orthodox or Protestant religions, those not watching television or listening to the radio, and married participants (Figure 6).

## Discussion

This study determined and predicted the pooled prevalence of current khat chewing practices and the factors influencing them among men in Ethiopia using a machine learning algorithm. Seven machine learning classifiers were trained on both balanced and imbalanced training datasets. Classification accuracy and AUC scores were used to compare the performance of the seven classification models. The XGBoost classifier outperformed the other classifiers in predictive modeling on both unbalanced and balanced training data, achieving an accuracy of 87% and an AUC score of 0.94. As a result, XGBoost was identified as the best predictive model, and further analysis was conducted after optimizing its hyperparameters. To date, no studies have used machine learning algorithms to predict khat chewing practices among men using these algorithms.

The pooled prevalence of khat chewing among men in Ethiopia from 2011 to 2016 was 32.79% (32.23–33.35). This finding is consistent with a study conducted among Dilla High School students (30). However, the prevalence observed in this study is lower than findings from studies conducted among university staff in Ethiopia (31), college and university students in Harar Town (32), Hossana Town, Ethiopia (15), professional drivers in Southwestern Saudi Arabia (33), and high school and preparatory school students in Ginnir town, Bale Zone, Southeast Ethiopia (34). This discrepancy may be due to the higher likelihood of khat exposure among these specific groups, whose work and lifestyle may increase their tendency to chew khat. Consequently, these populations exhibit a higher prevalence of khat chewing compared to the general male population in Ethiopia.

The findings of this study are higher than those of a previous study conducted among men in Ethiopia, particularly a further analysis of the 2016 DHS. This includes various studies, such as those involving students at Jima University, university students in Northwest Ethiopia, adults aged 15–49 years in Ethiopia, male adults in Ethiopia, youth in Ethiopia, a systematic and meta-review analysis among university students in Ethiopia, high school students in Eastern Ethiopia, medical students in Gondar town, and undergraduate students at Jimma University, Ethiopia (3, 12, 19, 20, 35–40). The majority of earlier studies focused on selected groups of respondents, such as students or teachers, or were based on small sample sizes. In addition, previous studies using the national survey were based on a single episode for analysis. Consequently, this may have resulted in a lower prevalence than that reported in the current study.

This study identified age, wealth index, region, religion, frequency of watching television, frequency of listening to the radio, age at first sexual encounter, place of residence, and number of living children as the most significant variables for predicting khat chewing practices among men.

The region has been identified as a predictor of khat chewing practices in Ethiopia among men aged 15 to 59 years, consistent with previously published studies (19, 20, 38). This can be explained by the fact that in certain regions, people consider khat chewing a normal part of life. Such regions include Somali, Afar, Benshangulgumz, and Harari. In Harar, khat consumption is deeply embedded in local culture and plays a key role in social, political, and spiritual practices, often involving communal gatherings. The khat industry also serves as a major economic driver, with many households depending on its cultivation and sale. In Dire Dawa, khat chewing is prevalent among students and workers, often linked to socialization and productivity, further solidifying its widespread use and social acceptance (5). In this region, khat chewing is regarded as a routine practice, akin to food and drink. This cultural difference across regions, especially regarding khat chewing in the eastern part of Ethiopia, is culturally accepted and viewed as a positive practice.

The Ethiopian government regulates khat through legal age limits, distribution controls, and export taxes; however, issues such as illegal taxation have required federal intervention. Health concerns, including increased blood pressure and heart rate, have been highlighted by the Ethiopian Public Health Institute to inform public health policies. Educational programs, particularly in high-consumption regions such as Harar, focus on life skills training to help students resist peer pressure. Regulation varies by region: while Harari, Oromia, and Somali enforce strict taxation and age limits, cultural acceptance in areas such as Harar and Dire Dawa leads to more lenient policies. Meanwhile, Amhara and Tigray prioritize health education over restrictions. Despite these efforts, khat remains widely consumed, especially in Addis Ababa, prompting ongoing public health initiatives to raise awareness and mitigate its risks (4, 41).

Similarly, religion has been identified as a significant determinant of khat chewing practices among men, consistent with earlier studies (3, 12, 15, 19, 30, 32, 35, 39). This could be explained by the fact that these religions may have various confounding factors that promote khat chewing. Traditionally, some followers accept khat chewing as a means to achieve maximum concentration during work and prayer. For instance, Muslim Ethiopians commonly consume khat during religious ceremonies such as Ramadan, holiday celebrations, and pilgrimages. It is also used in rituals, including singing, praying, and blessings. This perception highlights the association of khat consumption with Islamic identity. Research indicates that khat use among Muslim respondents is several times higher compared to that of their non-Muslim counterparts (15, 42, 43).

Similarly, this study revealed that an individual’s wealth index is a significant predictor of khat chewing practices, consistent with previously published studies (19, 44). Although khat chewing is often associated with wealthier groups due to habits formed during cultivation and business activities, it can also impose significant financial burdens on individuals and households. Regular consumption diverts substantial income from essential needs such as food, healthcare, and education, exacerbating financial hardships, especially for lower-income groups. The high cost of khat contributes to economic struggles, with many perceiving their financial difficulties as rooted in their consumption habits. Additionally, khat chewing sessions can last for hours, reducing productivity and work performance, ultimately leading to lower income and reinforcing poverty (45). Furthermore, individuals facing socioeconomic challenges may turn to khat as a coping mechanism for stress, further deepening their financial instability.

On the other hand, the frequency of watching television and listening to the radio has also been identified as a significant determinant of khat chewing practices, consistent with previously published studies (44, 46). The media plays a crucial role in influencing khat chewing behaviors within the population. Attitudes toward khat chewing are significantly influenced by media communication, and evidence indicates that exposure to media messages about khat chewing affects both the practice of khat chewing and its prevention.

Furthermore, this study revealed that age was a significant predictor of khat chewing practices, consistent with previously published studies (15, 20, 36, 37, 44, 46). Older men are more likely to chew khat, possibly because they experience stress, mood disturbances, and mental health issues more frequently than younger men, using khat as a coping mechanism. Similarly, this study found that place of residence is a significant predictor of khat chewing practices, consistent with previously published studies (36, 44).

Rural residents were less likely to chew khat, possibly due to a lack of exposure to different cultures, leading them to perceive the practice as wrong. Moreover, this study revealed that age at first sexual encounter was a significant predictor of khat chewing practices, suggesting that early sexual experiences contribute to breaking sociocultural norms and encountering stressful situations. Additionally, partners may have prior experience with khat chewing. Furthermore, this study highlighted that the number of living children is a significant predictor of khat chewing practices.

## Limitations and strengths of the study

This study’s primary strength lies in its use of large sample sizes and nationally representative data. Another key point is the application of a sophisticated statistical method (a machine learning technique) that revealed previously undiscovered relationships and patterns in the field. To determine the relative significance of each predictor and understand how each component contributed to the model’s predictions, the researchers employed various methodologies, including SHAP. This approach helped them understand how various factors affected the model’s predictions.

Despite its broad scope and large sample size, this study has limitations. For example, self-reporting is the primary method used to assess khat chewing practices. Consequently, there is a risk that social desirability bias may lead to underreporting.

## Conclusion

The prevalence of khat chewing in Ethiopia is high. The XGBoost classifier demonstrated superior predictive performance in identifying khat chewing practices compared to other machine learning models, achieving an accuracy of 87%, an AUC of 94, a precision of 86, a recall of 85, and an F1 score of 86. The model was evaluated using both a train-test split and 10-fold cross-validation to ensure robust performance, with both methods yielding comparable results. The application of SMOTE to balance the training data helped mitigate potential biases, further confirming the model’s reliability in predicting khat chewing behavior.

The findings from the SHAP analysis in the machine learning model provide a nuanced understanding of the key factors influencing khat chewing among men, offering deeper insights beyond traditional logistic regression analysis. This approach captures complex, nonlinear relationships and ranks variable importance based on predictive power. Accordingly, age, wealth index, region, religion, frequency of watching television, frequency of listening to the radio, age at first sex, residence, and number of living children are the most important factors in predicting khat chewing behavior. Unlike logistic regression, which assumes a linear relationship between predictors and outcomes, SHAP analysis reveals how individual variables contribute to predictions on a case-by-case basis, allowing for a more detailed interpretation of the interactions between multiple factors. These insights can guide more personalized and effective prevention strategies tailored to specific high-risk groups that traditional regression models might overlook.

To effectively prevent khat chewing, it is crucial to foster and promote rural community norms that discourage its use while extending these practices to urban areas where consumption is prevalent. Age-specific interventions targeting young and middle-aged men, alongside youth development programs, can provide healthier alternatives for socialization. Economic empowerment initiatives should be introduced to address the financial drivers behind khat use, particularly in high-prevalence regions. Strengthening family values through community marriage counseling and spouse-involvement programs can also play a role in reducing substance use. Additionally, khat prevention education should be integrated into reproductive health programs, considering the link between the age of first sexual encounter and khat consumption.

Religious leaders, especially from the Orthodox Church, should actively engage in delivering anti-khat messages through their sermons and teachings. Their insights can inform policy development, while alternative religious social events can serve as substitutes for khat-chewing gatherings. Educational and media campaigns should emphasize the harmful effects of khat, integrating these messages into school curricula as well as targeted TV and radio programs. Policy measures such as restricting khat sales near schools and religious institutions, enforcing community bylaws to discourage public consumption, and introducing alternative economic programs in rural areas can further reduce reliance on khat.

## Data Availability

The raw data supporting the conclusions of this article will be made available by the authors without undue reservation.

## Footnotes

^1^ https://dhsprogram.com/data/dataset_admin/index.cfm?CFID=1242368&CFTOKEN=c892a8da9855f981-8D71EDAA-BF68-5950-1D6BA7F0BB37D5E8
